# Empathy and emotion recognition in people with autism, first-degree relatives, and controls

**DOI:** 10.1016/j.neuropsychologia.2012.11.013

**Published:** 2012-11-19

**Authors:** E. Sucksmith, C. Allison, S. Baron-Cohen, B. Chakrabarti, R.A. Hoekstra

**Affiliations:** a Department of Life, Health and Chemical Sciences, The Open University, Milton Keynes MK7 6AA, UK; b Department of Psychiatry, Autism Research Centre, University of Cambridge, Cambridge, UK; c School of Psychology and Clinical Language Sciences, Centre for Integrative Neuroscience and Neurodynamics, University of Reading, Reading, UK

**Keywords:** Autism, Empathy, Emotion, Broader autism phenotype, Endophenotype

## Abstract

Empathy is the lens through which we view others’ emotion expressions, and respond to them. In this study, empathy and facial emotion recognition were investigated in adults with autism spectrum conditions (ASC; *N*=314), parents of a child with ASC (*N*=297) and IQ-matched controls (*N*=184). Participants completed a self-report measure of empathy (the Empathy Quotient [EQ]) and a modified version of the Karolinska Directed Emotional Faces Task (KDEF) using an online test interface. Results showed that mean scores on the EQ were significantly lower in fathers (*p* < 0.05) but not mothers (*p* > 0.05) of children with ASC compared to controls, whilst both males and females with ASC obtained significantly lower EQ scores (*p* < 0.001) than controls. On the KDEF, statistical analyses revealed poorer overall performance by adults with ASC (*p* < 0.001) compared to the control group. When the 6 distinct basic emotions were analysed separately, the ASC group showed impaired performance across five out of six expressions (happy, sad, angry, afraid and disgusted). Parents of a child with ASC were not significantly worse than controls at recognising any of the basic emotions, after controlling for age and non-verbal IQ (all *p* > 0.05). Finally, results indicated significant differences between males and females with ASC for emotion recognition performance (*p* < 0.05) but not for self-reported empathy (*p* > 0.05). These findings suggest that self-reported empathy deficits in fathers of autistic probands are part of the ‘broader autism phenotype’. This study also reports new findings of sex differences amongst people with ASC in emotion recognition, as well as replicating previous work demonstrating empathy difficulties in adults with ASC. The use of empathy measures as quantitative endophenotypes for ASC is discussed.

## 1 Introduction

Autism spectrum conditions (ASC) are neurodevelopmental in origin, and are characterized by difficulties with social interaction and communication, together with unusually restricted, repetitive behaviours and interests (APA, 2000; WHO, 1993). ASC involve a large number of behavioural manifestations that vary considerably across individuals and development. It is therefore important to test neurocognitive models that reduce these behaioural symptoms to a small number of underlying processes.

One of the earliest and most influential neurocognitive models for ASC is the theory of mind (ToM)/‘mind-blindness’ hypothesis. This states that the behaviour observed in ASC is due to difficulties representing the contents of one’s own and other people’s minds (Baron-Cohen, 1995). Successful social interaction requires the ability to attribute mental states to others in order to explain and predict their behaviour. Early studies assessing ToM in ASC and typically developing children primarily focused on the application and understanding of beliefs (Baron-Cohen, Leslie, & Frith, 1985; Leslie & Frith, 1988; Perner, Frith, Leslie, & Leekam, 1989), intentions (Phillips, Baron-Cohen, & Rutter, 1998) and pretence (Baron-Cohen, 1987; Leslie, 1987; Scott & Baron-Cohen, 1996). The ToM hypothesis can explain the social features of ASC but never set out to explain its non-social features. The hypothesis can also only explain the earliest symptoms of ASC by reference to simpler precursors of ToM, such as joint-attention and pretence (Pellicano, 2011). More recently, empathy has been proposed as a broader neurocognitive construct underlying the social and communicative difficulties observed in people with ASC (Baron-Cohen, 2002). Empathy extends the ToM hypothesis by not only focusing on the attribution of another person’s mental state but also on the capacity to respond to another’s mental states with an appropriate emotion (Baron-Cohen, 2002). It therefore includes both a cognitive component (identifying other people’s beliefs, desires, intentions etc.) and an affective component (responding to other people’s mental states with an appropriate emotion) (Baron-Cohen & Wheelwright, 2004; Chakrabarti & Baron-Cohen, 2006a).

The present study explores the hypothesis that the social communicative features of ASC entail empathy difficulties. This is tested using a self-report measure of empathy, the empathy quotient [EQ] (Baron-Cohen & Wheelwright, 2004). Self-report scales are useful in adulthood but one of their limitations is that a participant’s responses may not accurately reflect their true capabilities. Therefore, this study also includes a test of facial emotion recognition, as a performance measure.

Previous studies of the ability to recognize facial expressions of emotion in ASC have produced inconsistent results. Many studies have identified deficits in specific, negatively valenced expressions, including fear (Howard et al., 2000; Pelphrey et al., 2002), anger (Giola & Brosgole, 1988) and disgust (Golan, Baron-Cohen, & Hill, 2006) whilst other studies have identified impairments across all negative basic emotions (Ashwin, Chapman, Colle, & Baron-Cohen, 2006). Other studies have not found differences in basic emotion recognition performance in ASC (Adolphs, Sears, & Piven, 2001; Loveland et al., 1997; Rutherford & Towns, 2008). A review by Harms, Martin, and Wallace (2010) concluded that these discrepant findings were largely attributable to differences in IQ, task demands (static versus dynamic facial stimuli) and the types of dependent variables measured (electrophysiological/behavioural). Other studies have attributed the discrepant findings to variability in the intensity of emotions used as task stimuli (Law Smith, Montagne, Perrett, Gill, & Gallagher, 2010).

A proportion of ‘unaffected’ relatives of people with ASC exhibit milder features of the full autism phenotype. These traits, termed the ‘Broader Autism Phenotype’ (BAP) (Bolton et al., 1994), occur at behavioural, cognitive and neurophysiological levels. However, only a small number of features have consistently been found to occur frequently in the unaffected relatives of ASC probands. These include social communication difficulties and reduced performance on measures of social cognition (Sucksmith, Roth, & Hoekstra, 2011; Wheelwright, Auyeung, Allison, & Baron-Cohen, 2010). Previous studies of the BAP have included emotion recognition performance. Some of these have found first-degree relatives to exhibit milder difficulties in recognizing facial expressions (Losh et al., 2009; Palermo, Pasqualetti, Barbati, Intelligente, & Rossini, 2006; Wallace, Sebastian, Pellicano, Parr, & Bailey, 2010; but see Bölte & Poustka, 2003). To date, there have been no studies assessing whether the relatives of individuals with ASC self-report less empathy compared to a control group.

The primary aim of this study was to assess whether parents of children with ASC show reduced self-reported empathy, as well as emotion recognition difficulties, compared to IQ-matched controls, as part of the BAP. Second, we sought to replicate previous findings of difficulties with empathy and emotion recognition in adults with ASC. Finally, we tested if there are sex differences in each of the three groups (adult controls, parents of children with ASC, and in adults with ASC) on self-report and performance measures of empathy. Previous studies suggest significant sex differences in the general population for empathy measures, with females on average reporting higher empathy and outperforming males on performance-based tasks of empathy (Baron-Cohen & Hammer, 1997; Baron-Cohen & Wheelwright, 2004). Likewise, a small number of studies suggest sex differences within ASC itself on various behavioural measures (Bölte, Duketis, Poustka, & Holtmann, 2011; Lai et al., 2011), but this remains an under-researched area, largely due to difficulties in recruiting enough female participants with ASC. In our online study it was possible to recruit a relatively large sample of both males and females with a clinical ASC diagnosis.

## 2 Methods

### 2.1 Participants

Parents of children with an ASC diagnosis and adults with an ASC diagnosis were recruited from the Cambridge University Autism Research Centre volunteer database (www.autismresearchcentre.com). Recruitment of participants to this database has ethics approval from the Cambridge University Psychology Research Ethics Committee. During the registration process parents confirmed if they have a diagnosis of ASC themselves, and we excluded those who did. They also had to have at least one child with a diagnosis of ASC from a clinician based on DSM-IV or ICD-10 criteria. Adults with ASC confirmed that they had been diagnosed by an experienced clinician according to DSM-IV or ICD-10 criteria. Control participants were also recruited online, via a different portal (www.cambridgepsychology.com). During the registration process, control participants confirmed that they do not have an ASC diagnosis and that they were not the parent of a child with an ASC diagnosis. We excluded control participants with any other psychiatric diagnosis.

In total, 187 adult controls (93 males, 94 females), 310 parents of children with ASC (38 males, 272 females) and 329 adults with ASC (161 males, 168 females) completed the EQ. These groups did not significantly differ on non-verbal IQ (*p*=0.34) measured using an online adaptation of the Raven’s Progressive Matrices (RPM; Raven, Court, & Raven, 1996). After data cleaning and careful matching for non-verbal IQ (*p*=0.19), the following samples sizes were available for the KDEF test: 184 adult controls (92 males, 92 females) 297 parents (36 males, 261 females), and 314 adults with ASC (164 males, 150 females).

Approximately equal numbers of males and females were recruited in the control and ASC groups for both measures. In the parent group, there were more mothers than fathers on both measures, probably reflecting previous findings of higher response rates in females compared to males (Gosling, Vazire, Srivastava, & John, 2004). The mean age of participants completing each measure differed slightly across groups; the parents of children with ASC were older than both controls and adults with ASC. Nevertheless, the range of ages in the ASC parent group was similar to controls and adults with ASC (ASC parents: 24–61 years, ASC: 16–70 and Controls: 19–65). Table 1 displays descriptive data for the three groups of participants that completed the EQ and KDEF, including sample sizes, mean ages and IQ scores.

### 2.2 Materials and procedure

After registering online and consenting to take part in research, participants were asked to complete the different measures in their order of preference. These included the Empathy Quotient (EQ; Baron-Cohen & Wheelwright, 2004) which consists of 40 items, where participants respond to each item using a 4 point Likert scale (‘strongly agree’, ‘slightly agree’, ‘slightly disagree’ and ‘strongly disagree’). An empathic response to an item is given a score of ‘1’ or ‘2’ depending on the strength of the response. Twenty-one out of the forty scored items are reversed to avoid response biases. Other responses are given a score of ‘0’. Scores on each item are summed providing a total score between 0 and 80. There were no missing values.

The EQ has excellent test-retest reliability (*r*=0.97, *p* < 0.001; Baron-Cohen & Wheelwright, 2004) and good construct validity, correlating positively with a performance-based measure of social cognition (the ‘Eyes’ task; *r*=0.294, *p* < 0.05; Lawrence, Shaw, Baker, Baron-Cohen, & David, 2004). It also has high internal consistency (Cronbach’s alpha=0.92; Baron-Cohen & Wheelwright, 2004). Currently the most comprehensive assessment of the dimensionality of the EQ using a Rasch and confirmatory factor analysis suggests that the EQ is a unidimensional measure (Allison, Baron-Cohen, Wheelwright, Stone, & Muncer, 2011).

**Fig. 1:**
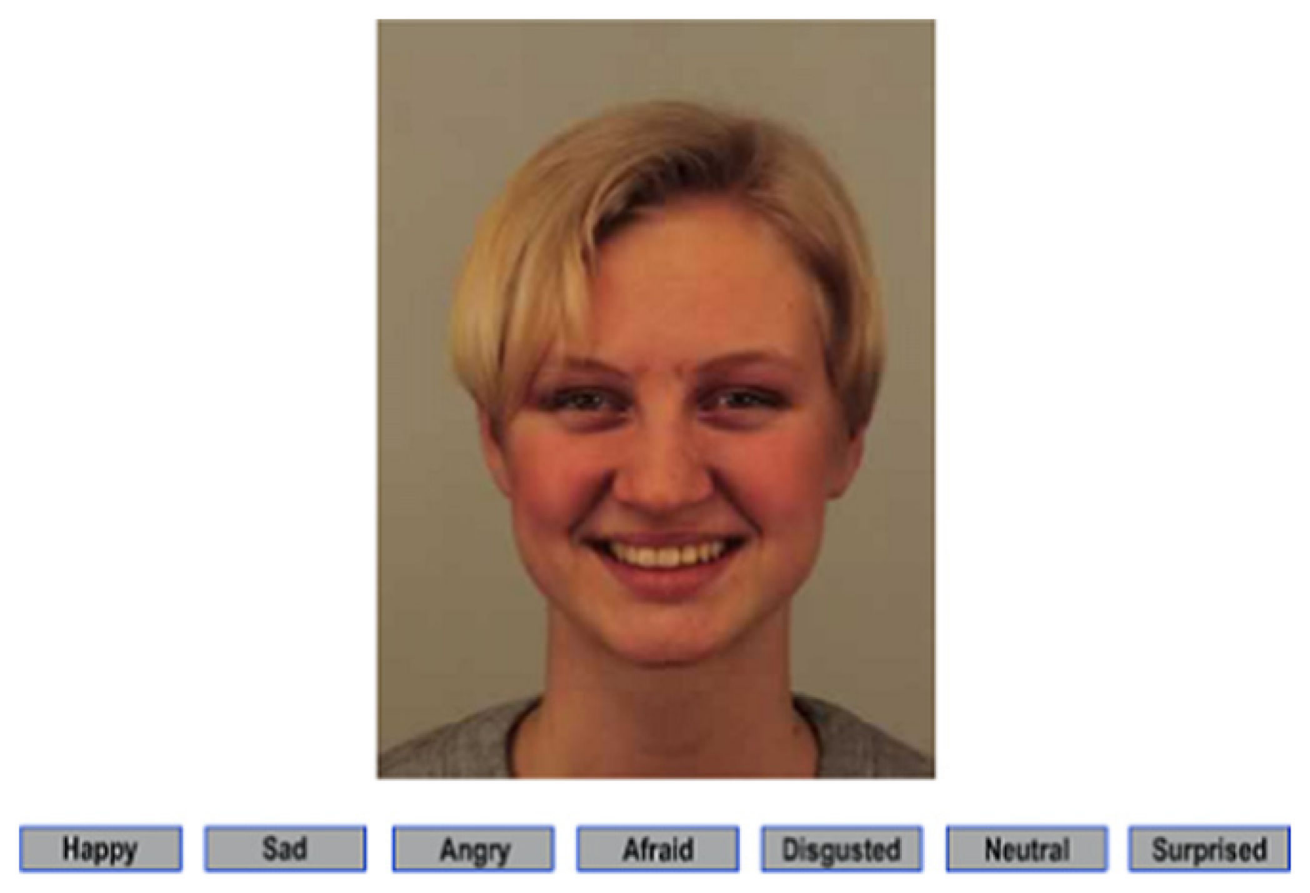
Example of Stimuli used in the KDEF (Lundqvist et al., 1998). KDEF; Karolinska directed emotional faces task. KDEF stimulus ID: happy af28.

Participants also completed a modified version of the Karolinska directed emotional faces task (KDEF; Lundqvist, Flykt, & Ohman, 1998) using the online test interface. Participants were shown 140 photographs of people’s faces expressing one of six ‘basic’ emotions (happy, sad, angry, afraid, disgusted and surprised) as well as a neutral expression (see Fig. 1). There were 20 photographs in total for each expression. For each photograph, participants were asked to select which of the seven words described the emotion being expressed. Participants were told they had 20 s to respond to each photograph and they must answer as quickly and accurately as possible. Results provide an accuracy score and response time (for correct trials only) for each facial expression of emotion. The stimuli used in the KDEF have been validated on emotional content, intensity and arousal and have good test–retest reliability (Goeleven, De Raedt, Leyman, & Verschuere, 2008). Furthermore, the KDEF stimuli set have good ecological validity, unlike schematic or computerized faces (see Supplementary material for the stimuli ID codes selected for this task).

All data were rigorously checked prior to the data analyses. Twenty-two data points were identified as outliers (> 3 standard deviations from the group mean) and so were removed from the data set, resulting in the final sample size of 314 adults with ASC, 297 parents and 184 control participants.

Finally, participants used the online test interface to complete an online adaptation of the RPM, a measure of non-verbal intelligence (Raven et al., 1996). The RPM consists of 60 items displaying geometric designs of varying complexity that contain a missing piece. Participants had to choose from a selection of designs to complete the pattern. Performance on the online RPM was used so that groups could be matched on non-verbal IQ; this ensures that the relationship between group status and the empathy/emotion recognition measures is undistorted by non-verbal IQ and that any significant differences found reflect selective difficulties in behaviour/cognition. RPM accuracy score was also used as a covariate in data analyses to remove any covariance from the outcome measures that could be attributed to variation in non-verbal cognitive ability.

### 2.3 Statistical analyses

Adults with ASC, parents of children with ASC and the control group were compared on mean EQ scores using a univariate analysis of covariance (ANCOVA) with non-verbal IQ and age used as covariates. Previous studies have reported sex-specific expression of the BAP (Constantino et al., 2006; Happé, Briskman, & Frith, 2001) and sex differences on measures of empathy (Baron-Cohen & Wheelwright, 2004), so sex was also used as a between-subjects factor in the data analyses.

For the KDEF, two dependent variables were analysed. First, accuracy was used, in line with previous research on facial emotion recognition in ASC (Ashwin et al., 2006; Bölte & Poustka, 2003). Second, ‘accuracy-adjusted response time’ was used which is likely to be a more sensitive measure as it controls for a potential speed-accuracy trade-off (see Mevorach, Humphreys, & Shalev, 2006 and Sutherland & Crewther, 2010 for similar approaches). Accuracy scores showed high ceiling effects, with distributions significantly deviating from the normal distribution. Therefore, non-parametric Kruskal–Wallis tests were carried out on accuracy scores for each emotion, with group used as the fixed factor. For emotions that showed significant differences, planned follow-up Mann–Whitney *U* tests were carried out between ASC parents and controls and between ASC adults and controls.

Accuracy-adjusted response times were calculated for each emotion by dividing the mean response time for correct items by the fraction of items answered correctly. This ratio provides a degree of adjustment for potential speed-accuracy tradeoffs. Adults with ASC, parents of children with ASC and the control group were compared on this dependent variable using a mixed analysis of covariance (ANCOVA). This test was used to compare groups on overall mean accuracy-adjusted response time across all emotions. Follow up ANCOVAs with planned contrasts were then carried out to compare groups on each emotion separately. In these analyses, sex was again included as a fixed factor and non-verbal IQ and age used as covariates.

## 3 Results

### 3.1 Self-rated empathy

**Fig. 2:**
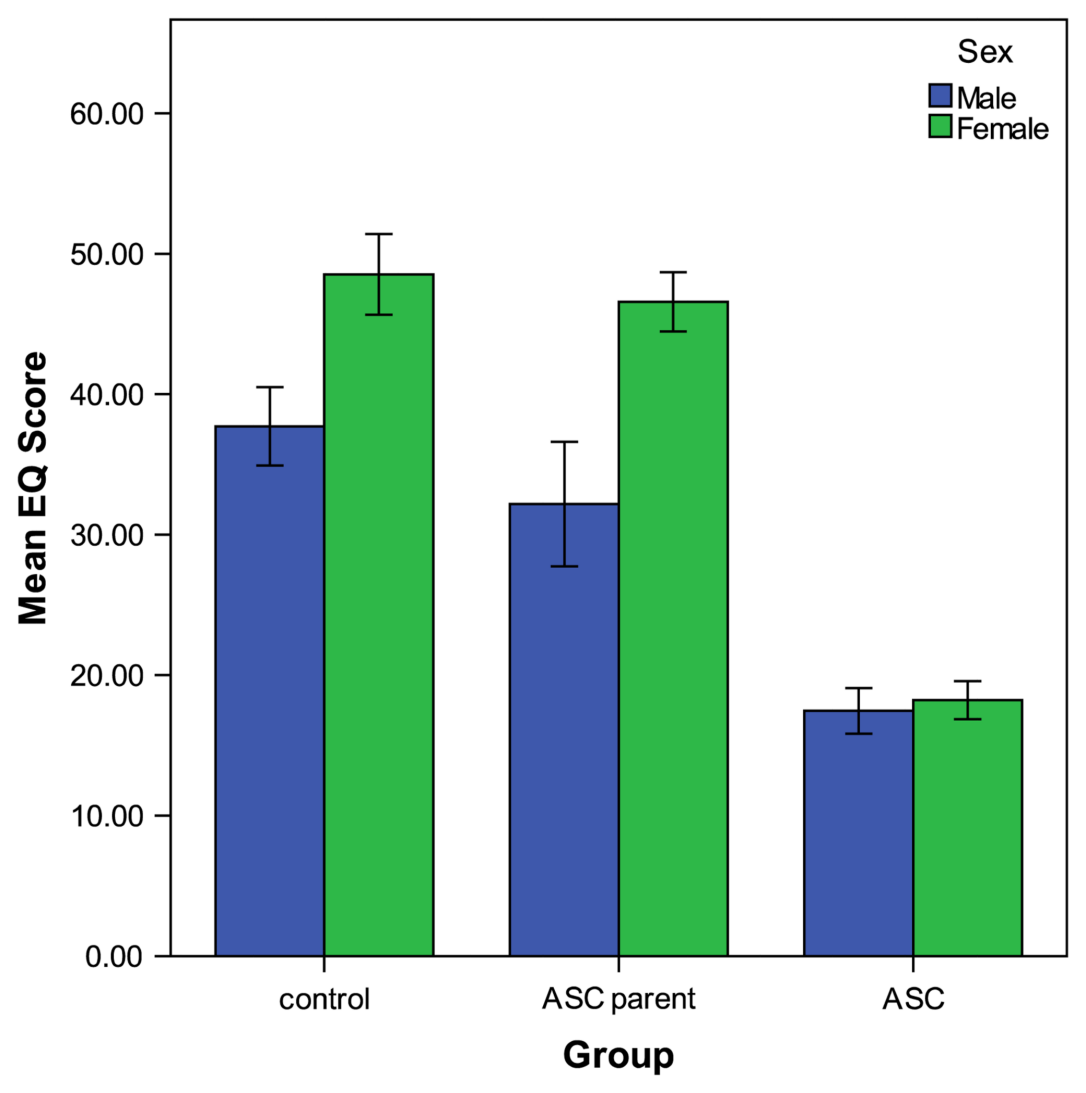
Main effects of group and sex on mean EQ score. EQ; empathy quotient. Error bars depict the 95% confidence intervals.

Table 2 shows the mean EQ scores, standard deviations and available sample sizes for each group, separated by gender. A group × sex ANCOVA with age and non-verbal IQ as the covariates showed that age did not have a significant effect on mean EQ score (*F*(1,818)=0.25, *p*>0.05), whilst non-verbal IQ was significantly related to mean EQ score (*F*(1,818)=10.59, *p* < 0.01; Pearson’s correlation coefficient *r*=0.11, indicating a small effect size and thus a modest positive association between empathy and non-verbal IQ). Results also revealed a significant main effect of group (*F*(2,818)=242.60, *p* < 0.001). Contrast analyses suggested that the mean EQ score was significantly lower in adults with ASC (*p* < 0.001, *r*=0.51) compared to the control group. The ANCOVA also revealed a significant main effect of sex (*F*(1,818)=57.06, *p* < 0.001, *r*=0.30), with females obtaining higher scores than males. A significant interaction effect between group and sex on mean EQ score (*F*(2,818)=14.64, *p* < 0.001) was seen, suggesting that group effects are different for males and females (see Fig. 2). Results from subsequent sex-specific ANCOVAs confirmed that both males and females with ASC reported significantly lower EQ scores on average than controls (*p* < 0.001. See Table 2 for mean scores). However, contrasts confirmed that fathers, but not mothers, of children with ASC reported a significantly lower mean EQ score compared to sex-specific controls (fathers: *p* < 0.05, *r*= 0.32; mothers: *p* = 0.21). Results from group-specific ANCOVAs confirmed that there was a non-significant difference between male and female EQ scores in adults with ASC (*p*=0.40) but significant differences between males and females in the control group (*p* < 0.001, *r*=0.37) and the ASC parent group (*p* < 0.001, *r*=0.07). This suggests that the significant group × sex interaction is partially caused by sex differences in mean EQ score amongst controls and ASC parents, whereas sex differences are absent in individuals with ASC (see Fig. 2).

### 3.2 Emotion recognition

#### 3.2.1 Accuracy

Table 2 displays the descriptive data for performance on the KDEF task, which includes accuracy and accuracy-adjusted response time. Kruskal–Wallis tests were carried out on accuracy scores for each emotion separately. These revealed a significant effect of group on four out of six basic emotions (happy, angry, afraid and disgust; *p* < 0.001) as well as the neutral expression (*p* < 0.05). Follow up Mann–Whitney *U* tests indicated that, compared to controls, adults with ASC were significantly less accurate at identifying these emotions (happy; *p* < 0.05, angry; afraid; disgust; *p* < .001) and at identifying neutral expressions (*p* < 0.05). Conversely, no significant differences were found between ASC parents and controls on these expressions (all *p* > 0.05).

#### 3.2.2 Accuracy-adjusted response time

**Fig. 3:**
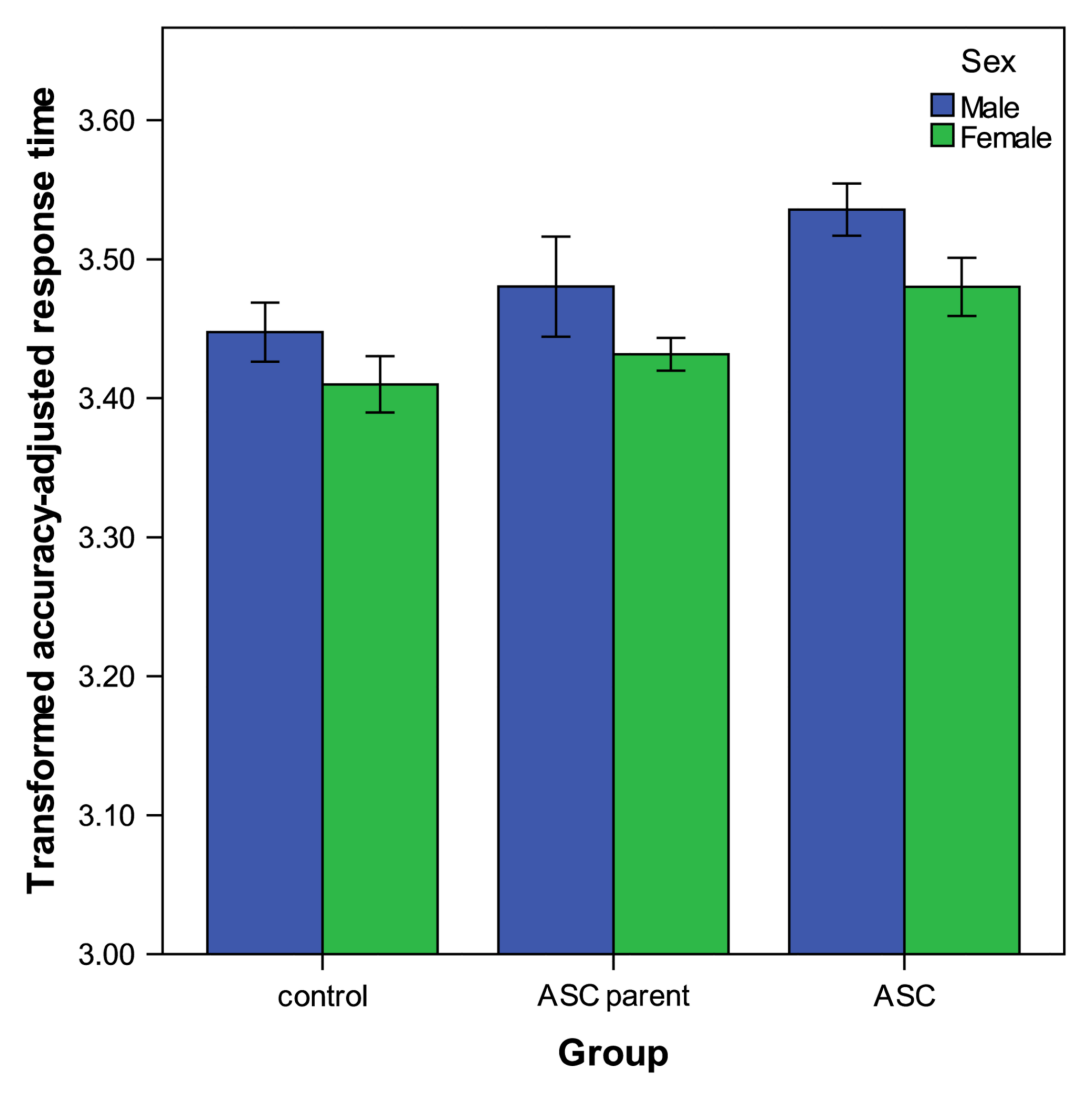
Main effects of group and sex on overall accuracy-adjusted response times on the KDEF. KDEF; Karolinska directed emotional faces task. Mean accuracy-adjusted response times displayed are across all facial expressions of emotion. Error bars depict 95% confidence intervals.

Accuracy-adjusted response times were logarithmically transformed to enable the use of parametric tests of statistical inference. After transformation the distribution was approximately normal in all groups. A mixed analysis of covariance (ANCOVA) was carried out on mean accuracy-adjusted response times for each emotion, with group and sex as fixed factors and non-verbal IQ and age as the covariates. This revealed a significant main effect of group (*F*(2,787)=40.83, *p* < 0.001) and of sex (*F*(1,787)=17.43, *p* < 0.001, *r*=0.15). The group × sex interaction effect failed to reach significance (*p* > 0.05), whilst the covariates (non-verbal IQ and age) had significant effects on accuracy-adjusted response time (non-verbal IQ; *F*(1,787)=9.54, *p* < 0.01, age; *F*(1,787)=16.43, *p* < 0.001). Contrast analyses indicated that adults with ASC, but not ASC parents, had a significantly higher overall mean accuracy-adjusted response time compared to controls (ASC adults; *p* < 0.001, ASC parents; *p* > 0.05). Contrasts also indicated significant differences in overall mean accuracy-adjusted response time between males and females across the three groups. Results from group-specific ANCOVAs indicated that the sex differences in accuracy-adjusted response time were significant in the control group (*p* < 0.01, *r*=0.19), ASC parent group (*p* < 0.05, *r*=0.14) and ASC group (*p* < 0.001, *r*=0.21), with females outperforming males across all groups (see Fig. 3).

**Fig. 4:**
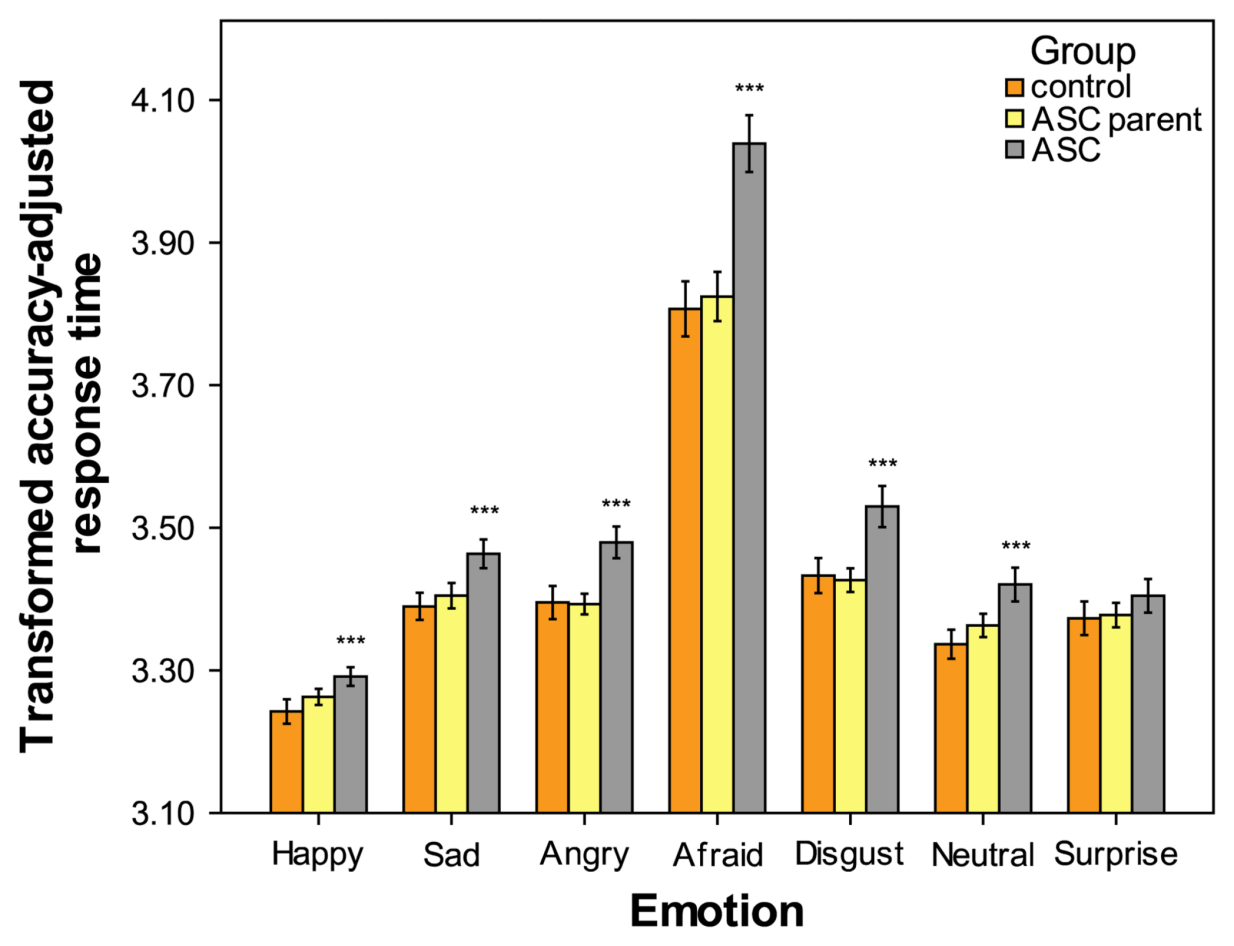
Main effect of group on mean accuracy-adjusted response times for separate facial expressions of emotion on the KDEF. KDEF; Karolinska directed emotional faces task. Significant differences between control and experimental groups denoted by the asterisks: ***p < 0.001. Error bars depict the 95% confidence intervals.

Fig. 4 displays the main effect of group on accuracy-adjusted response times for individual facial expressions of emotion. Follow up ANCOVAs were carried out on mean accuracy-adjusted response times for each emotion and the neutral expression, with group and sex as fixed factors and non-verbal IQ and age as the covariates. These analyses revealed a significant main effect of group on accuracy-adjusted response time for five emotions and the neutral expression (happy; sad; angry; afraid; disgust; neutral; *p* < 0.001). There was also a significant main effect of sex on accuracy-adjusted response time for five emotions (disgust; surprise; *p* < 0.001, sad; angry; *p* < 0.01, happy; *p* < 0.05). The non-verbal IQ covariate had a significant effect on the accuracy-adjusted response time for three facial expressions (afraid; *p* < 0.001, angry; disgust; *p* < 0.05), whilst the age covariate had a significant effect on the accuracy-adjusted response time for four facial expressions (happy; sad; neutral; *p* < 0.001, surprise; *p* < 0.01). There were no significant group × sex interactions (all *p*>0.05). Contrast analyses indicated that the accuracy-adjusted response times of adults with ASC were significantly higher than the control group on five emotions and the neutral expression (happy; sad; angry; afraid; disgust; neutral; *p* < 0.001). These contrasts also indicated that there were no significant differences between parents of children with ASC and controls on accuracy-adjusted response times for each facial expression (all *p*>0.05).

#### 3.2.3 Correlations with EQ score

Lastly, the correlation between self-reported empathy and emotion recognition was explored in all three groups. Mean EQ scores and mean KDEF accuracy-adjusted response times were negatively correlated (ASC: *r*= −0.16, *p* < 0.01, ASC parents: *r*= −0.15, *p* < 0.01 and Controls: *r*= −0.15, *p* < 0.05). These significant correlations suggest that the EQ and KDEF measure modestly overlapping constructs, such that people with relatively low self-rated empathy score somewhat lower on the performance test for emotion recognition.

## 4 Discussion

This study investigated empathy and facial emotion recognition in adults with ASC and in first-degree relatives (parents) of children with ASC. The evidence supports a broader autism phenotype (BAP) for self-rated empathy in fathers of children with ASC, but not for basic facial emotion recognition in parents of children with ASC. We also replicated previous studies reporting empathy and emotion recognition difficulties in adults with ASC, and found evidence for a difference between males and females with ASC on emotion perception. Each of these findings is discussed below.

Fathers but not mothers of children with ASC self-reported lower empathy than controls on the empathy quotient (EQ). This suggests that lower self-reported empathy may be a reliable feature of the BAP in fathers only. Further research is needed to assess whether this sex-specific finding generalizes to other relatives, e.g., to brothers but not sisters of individuals with ASC. Some previous studies have suggested that certain aspects of the BAP may be especially prevalent in male relatives (Constantino et al., 2006). This study is the first to explore self-reported empathy in parents of a child with ASC. Equally, further research is needed to test if the absence of a self-reported empathy deficit in mothers is because they are over-estimating their true empathy level.

When analyzing facial emotion recognition using a sensitive measure of performance (accuracy-adjusted response time), parents of children with ASC were not significantly poorer than IQ-matched controls at identifying the six basic facial expressions of emotion. These results do not support the notion that there is a BAP for basic emotion recognition, in contrast to some previous studies (Palermo et al., 2006; Smalley & Asarnow, 1990; Wallace et al., 2010). One possible reason for these discrepant findings is that the measure of basic emotion recognition used here was not sensitive enough to detect subtle differences in basic emotion recognition in ASC relatives. Whilst the dependent variable used included a sensitive measure of emotion recognition performance (accuracy-adjusted response time), the KDEF stimuli comprise high intensity, ‘full blown’ emotions – exaggerated facial expressions – that were relatively easy to identify in non-clinical samples. Making emotional expressions more subtle would have increased task difficulty and may have increased the power to detect subtle differences in emotion recognition ability. Our previous study used the ‘Reading the Mind in the Eyes’ (Eyes) test that requires emotion recognition from just the eye region of the face and involves emotions beyond the basic ones. On the Eyes test, both mothers and fathers of children with ASC showed deficits (Baron-Cohen & Hammer, 1997). In clinical samples of ASC emotion recognition deficits have also emerged more clearly when using lower intensity stimuli (Law Smith et al., 2010).

A second possible reason for these discrepant findings is that mild difficulties in basic emotion recognition performance may be ‘compensated’ in parents of children with ASC. Evidence for cognitive compensation has been detected in first-degree relatives using neuroimaging techniques: at a neural level Spencer et al. (2011) found that unaffected siblings of children with ASC, showed reduced neural response (in multiple brain regions including the fusiform face area and superior temporal sulcus) to happy but not fear faces. These neurophysiological differences in siblings were seen despite non-significant differences in performance on the facial emotion recognition task. Understanding what occurs in such examples of ‘compensation’ will be important in future work.

A third finding from this study relates to adults with ASC. There was a significant sex difference in adults with ASC on the emotion recognition task, females with ASC performing significantly better than males. This contrasts with results on the EQ that did not show significant sex differences in adults with ASC. This suggests that females with ASC may perform better than males with ASC at tests of social cognition, despite having comparably low levels of self-reported empathy.

A number of different interpretations may account for these findings. Females’ low self-reported empathy may be more related to difficulties that extend beyond basic emotion recognition which were not analysed here (e.g., more advanced theory of mind). Alternatively, their low self-reported empathy may reflect higher social expectations on females in the real world. If typical females are expected to be better at empathy than males, this may cause females with ASC to report their empathy problems to a greater degree than males. Finally, these results may reflect greater cognitive compensation in females with ASC. Perhaps as a result of greater social expectations and greater motivation to integrate into social groups, females with ASC work harder to compensate for their problems by developing cognitive strategies to improve their social skills. Thus, females with ASC may have a heightened self-awareness of their social difficulties as a result of being more able than males with ASC to read the emotions of others. This interpretation is consistent with previous studies which find that people with ASC who display stronger intellectual and emotional capabilities perceive themselves as less socially competent than people with ASC who possess less emotional understanding (Capps, Sigman, & Yirmiya, 1995).

To date, only a small number of studies have investigated behavioural differences between males and females with ASC. Similar to the findings reported here Lai et al. (2011) found higher levels of autistic traits in females with ASC compared to males on a self-rating scale (the Autism Spectrum Quotient [AQ]; Baron-Cohen, Wheelwright, Skinner, Martin, & Clubley, 2001) but fewer social-communication difficulties on an observational measure (the Autism Diagnostic Observational Schedule [ADOS] (Lord et al., 2000)). Further studies are needed to confirm these findings and to test these different explanations.

In addition, the present study replicates previous results showing empathy and emotion recognition in people with ASC. First, empathy difficulties were detected in adults with ASC on the EQ. Like previous studies (Baron-Cohen & Wheelwright, 2004), this study found sex differences in the control group, with typical females reporting significantly higher empathy than males. Likewise, mothers of children with ASC reported significantly higher empathy than fathers of children with ASC. The present study also replicates previous reports of emotion recognition difficulty in adults with ASC (Ashwin et al., 2006; Bölte & Poustka, 2003). However, this study analysed performance on each emotion by taking into account accuracy and response time, and found that adults with ASC have difficulties recognizing both positive (happy) and negative emotions. Difficulties were found across a wider range of basic emotions than reported in previous studies that use smaller sample sizes (Ashwin et al., 2006; Pelphrey et al., 2002). It is possible that very large sample sizes are needed in order to have sufficient power to detect performance differences for specific facial expressions of emotion (e.g., happy and sad expressions).

In addition, many previous studies of facial emotion recognition only examine accuracy as a measure of performance, which is susceptible to ceiling effects and therefore less sensitive to pick up subtle differences in ability. Response time is important because there is strong evidence to suggest that the processing of social information takes longer in individuals with an ASC, perhaps as a result of differences in connectivity patterns within and between structures in the ‘social brain’ (Brothers, 1990; Isler, Martien, Grieve, Stark, & Herbert, 2010; Minshew & Williams, 2007). There is also evidence to suggest that milder but similar alterations in brain connectivity can be found in the first-degree relatives of autistic probands (Belmonte, Gomot, & Baron-Cohen, 2010; Spencer et al., 2011). Therefore, using a weighted response time measure for social cognition tasks may reveal important subtle differences in cognition between autistic probands, parents and controls, which may not be picked up by accuracy measures alone.

The present study implicates the use of empathy measures as potential endophenotypes for autism. Instead of focusing molecular genetic studies on finding genes associated with clinical diagnoses, studies focusing on endophenotypes may provide measures that are ‘upstream’ in the causal pathways from genes to clinical diagnosis (Chakrabarti et al., 2009; Gottesman & Gould, 2003). Since both the EQ and KDEF are quantitative measures, these instruments can quantify the heterogeneity in ASC, and may therefore help improve power to detect significant effects, especially for common genetic variants associated with ASC, for which the results have so far been inconsistent (Abrahams & Geschwind, 2008; Freitag, Staal, Klauck, Duketis, & Waltes, 2010; Holt & Monaco, 2011). However, this study suggests that a more subtle test of basic facial emotion recognition is required for first-degree relatives of children with ASC, rather than the task used in this current study, which involved high intensity emotional stimuli.

Facial emotion recognition could be a plausible candidate as an endophenotype for ASC. The ability to recognize basic facial expressions appears very early in life (Field, Woodson, Greenberg, & Cohen, 1982; Walker-Andrews, 1997; Walden & Ogan, 1988), is universal across cultures (Ekman & Friesen, 1971) and is acquired in closely related animal species (Darwin, 1872/2009). Therefore, it can be hypothesized that this simpler phenotype lies closer to the genes than the behavioural impairments characterizing ASC using DSM-IV criteria. Likewise, empathy as a trait may be a simpler phenotype than ASC (Baron-Cohen, 2009; Chakrabarti, Bullmore, & Baron-Cohen, 2006b).

Currently, only a few studies have tested empathy and emotion recognition as endophenotypes for ASC. For example, a functional MRI study of emotion recognition in children with ASC and their siblings has implicated a neuroimaging endophenotype for responses to happy (versus neutral) faces (Spencer et al., 2011). Likewise, a study investigating the neural correlates of empathizing has also suggested that the EQ may constitute a useful endophenotypic parameter for studying ASC (Chakrabarti et al., 2006b). Further studies are needed to replicate the results reported here, as well as exploring components of empathy beyond the recognition of basic emotions in people with ASC and their first degree relatives (Decety & Moriguchi, 2007).

There are a number of limitations to acknowledge in this study. First, although all participants in the ASC group reported a clinical diagnosis of ASC, these diagnoses could not be verified because data were collected online. However, Lee et al. (2010) provide evidence to suggest that registering diagnoses of ASC using an online registry of families is accurate. Lee et al. sampled families registered on an online database called the Interactive Autism Network (IAN) and phenotyped 107 children with a registered online diagnosis. 99% of this sample was ASC positive using the ADI-R and 93% was ASC positive on both the ADI-R and ADOS/expert clinician observation. It is therefore reasonable to assume that registered online diagnoses for this study are sufficiently reliable, especially in the parent group.

The online study design used in this study also had significant advantages. It enabled collection of much larger sample sizes than those previously on empathy and emotion recognition in people with ASC and their first-degree relatives (Baron-Cohen & Wheelwright, 2004; Baron-Cohen & Hammer, 1997; Bölte & Poustka, 2003; Wallace et al., 2010). Therefore, this study had greater power to detect differences that may not have been picked up in previous investigations looking at similar theoretical constructs. Furthermore, the online measures are completed by people in their own time in the comfort of their own home. This makes the study less stressful than face-to-face testing and may therefore be more valid.

The current study did not include a clinical control group. We cannot therefore exclude the possibility that the lower empathy scores in fathers of children with ASC was due to non-genetic factors associated with caring for a child with special needs. Further studies using a clinical control group are needed to rule out this possibility. Moreover, there were subtle age differences between groups, with parents of children with ASC being somewhat older than the ASC and control groups. Previous studies have reported significantly reduced performance on tests of emotion recognition with increasing age in adulthood (Calder et al., 2003, Montagne, Kessels, De Haan, & Perrett, 2007). It is therefore important to control for age in data analysis. The sample size was also comparatively small for fathers of children with ASC, but even with this sample size we were able to detect a significant group effect for fathers of a child with ASC. Power problems due to the relatively small group of fathers are therefore unlikely to play a role.

This investigation used a self-report measure of empathy. Some participants may experience difficulty judging their own empathy, so it would be of interest in future studies to include a measure of empathy rated by others. Ideally, multiple raters would be included to assess empathy (Bartels, Boomsma, Hudziak, van Beijsterveldt, & van den Oord, 2007).

In summary, this study provides support for low self-reported empathy in ASC fathers compared to IQ-matched controls, but no evidence for basic facial emotion recognition difficulties in either parent of a child with ASC. These mild empathy difficulties in ASC fathers confirm earlier studies (Baron-Cohen & Hammer, 1997) and echo the more pronounced deficits found in adults with a clinical ASC diagnosis, who self-reported significantly lower empathy than controls and were also significantly worse at identifying five basic facial expressions of emotion. These findings implicate empathy-related traits as candidate endophenotypes for ASC which could help to elucidate the genetic and biological pathways underlying clinical ASC.

## Supplementary Material

Supplementary data associated with this article can be found in the online version at http://dx.doi.org/10.1016/j.neuropsychologia.2012.11.013.

Supplementary Material

## Figures and Tables

**Table 1:** Descriptive data for group analysis of the EQ and KDEF^a^.

	EQ			KDEF		
	*N*	Mean age (SD)	Mean non-verbal IQ (SD)	*N*	Mean age (SD)	Mean non-verbal IQ (SD)
Control	187	34.3 (10.76)	52.7 (3.58)	184	34.4 (10.84)	52.7 (3.64)
ASC parent	310	41.0 (6.34)	52.1 (3.56)	297	41.0 (6.43)	52.1 (3.46)
ASC	329	35.5 (11.03)	52.3 (4.24)	314	35.7 (11.25)	52.5 (4.11)

a EQ; empathy quotient, KDEF; Karolinska directed emotional faces task.

**Table 2:** Descriptive data for group analysis of the EQ and performance on the KDEF, separated by gender^a^.

	Males			Females		
	Control	ASC parent	ASC	Control	ASC parent	ASC
**EQ**						
*N*	93	38	161	94	272	168
Mean score (SD)	37.7 (13.5)	32.2 (13.5)	17.5 (10.5)	48.5 (14.1)	46.6 (17.7)	18.2 (8.9)
**KDEF**						
*N*	92	36	164	92	261	150
Mean accuracy per emotion (/20) (SD)	17.49 (1.18)	17.34 (1.38)	16.60 (1.80)	17.80 (1.21)	17.71 (1.03)	16.70 (1.76)
Mean ART (ms) per emotion (SD)	2885.44 (745.14)	3113.44 (794.68)	3577.71 (1091.95)	2637.13 (621.80)	2774.75 (708.09)	3168.45 (1071.96)

a EQ; empathy quotient, KDEF; Karolinska directed emotional faces task, ASC; autism spectrum conditions, ART; accuracy-adjusted response time.
