# Supplementary material for: Empathy and emotion recognition in people with autism, first-degree relatives, and controls
Source: Neuropsychologia. Author manuscript; Available in PMC 2019 Jan 24. (PMC6345368; doi:10.1016/j.neuropsychologia.2012.11.013)
Supplement: Supplementary Material [file NIHMS81073-supplement-Supplementary_Material.doc]

**Supplementary Material**

*Materials and procedure*

The stimuli ID codes that were selected for the modified Karolinska Directed Emotional Faces task are listed as follows: Happy (af20, af22, af24, af26, af28, af30, af32, af33, am25, bf06, bf25, bm06, bm07, bm08, bm20, bm21, bm23, bm26, bm28, bm35), Sad (af02, af03, af07, af09, af10, af11, af13, af20, af26, am04, am05, am13, am14, am19, am23, am32, bf14, bm01, bm12, bm17), Angry (af06, af19, af23, af25, af29, af31, af33, am05, am08, am10, am17, am28, am31, bf15, bf17, bf26, bm07, bm15, bm21, bm34), Afraid (af01, af02, af07, af14, af16, af21, af31, af32, am04, am05, am23, am35, bf09, bf22, bm03, bm11, bm13, bm17, bm24, bm34), Disgust (af10, af13, af16, af17, af19, af21, af23, am02, am12, am13, am18, am24, am34, bf12, bf14, bf32, bm05, bm07, bm10, bm11), Neutral (af06, af07, af11, af14, af17, af18, af19, af29, af30, af34, am04, am06, am07, am08, am10, am13, am21, am25, am31, am33) and Surprise (af02, af14, af19, af20, af24, am02, am24, am31, am34, bf03, bf10, bf22, bf28, bf30, bm03, bm05, bm06, bm11, bm12, bm16).
